# Intensity and Dose of Neuromuscular Electrical Stimulation Influence Sensorimotor Cortical Excitability

**DOI:** 10.3389/fnins.2020.593360

**Published:** 2021-01-15

**Authors:** Ainhoa Insausti-Delgado, Eduardo López-Larraz, Jason Omedes, Ander Ramos-Murguialday

**Affiliations:** ^1^Institute of Medical Psychology and Behavioral Neurobiology, University of Tübingen, Tübingen, Germany; ^2^International Max Planck Research School (IMPRS) for Cognitive and Systems Neuroscience, Tübingen, Germany; ^3^IKERBASQUE, Basque Foundation for Science, Bilbao, Spain; ^4^Bitbrain, Zaragoza, Spain; ^5^Instituto de Investigación en Ingeniería de Aragón (I3A), Zaragoza, Spain; ^6^Departamento de Informática e Ingeniería de Sistemas (DIIS), University of Zaragoza, Zaragoza, Spain; ^7^Neurotechnology Laboratory, TECNALIA, Basque Research and Technology Alliance (BRTA), Donostia-San Sebastián, Spain

**Keywords:** neuromuscular electrical stimulation (NMES), electroencephalography (EEG), afferent cortical activation, sensorimotor oscillatory rhythm, artifact removal

## Abstract

Neuromuscular electrical stimulation (NMES) of the nervous system has been extensively used in neurorehabilitation due to its capacity to engage the muscle fibers, improving muscle tone, and the neural pathways, sending afferent volleys toward the brain. Although different neuroimaging tools suggested the capability of NMES to regulate the excitability of sensorimotor cortex and corticospinal circuits, how the intensity and dose of NMES can neuromodulate the brain oscillatory activity measured with electroencephalography (EEG) is still unknown to date. We quantified the effect of NMES parameters on brain oscillatory activity of 12 healthy participants who underwent stimulation of wrist extensors during rest. Three different NMES intensities were included, two below and one above the individual motor threshold, fixing the stimulation frequency to 35 Hz and the pulse width to 300 μs. Firstly, we efficiently removed stimulation artifacts from the EEG recordings. Secondly, we analyzed the effect of amplitude and dose on the sensorimotor oscillatory activity. On the one hand, we observed a significant NMES intensity-dependent modulation of brain activity, demonstrating the direct effect of afferent receptor recruitment. On the other hand, we described a significant NMES intensity-dependent dose-effect on sensorimotor activity modulation over time, with below-motor-threshold intensities causing cortical inhibition and above-motor-threshold intensities causing cortical facilitation. Our results highlight the relevance of intensity and dose of NMES, and show that these parameters can influence the recruitment of the sensorimotor pathways from the muscle to the brain, which should be carefully considered for the design of novel neuromodulation interventions based on NMES.

## Introduction

Neuromuscular electrical stimulation (NMES) is an electrophysiological technique that consists of applying electrical currents on the skin to depolarize motor and sensory nerves beneath the stimulating electrodes (Bergquist et al., 2011). NMES has been used as a neuroscientific tool to study sensorimotor neural mechanisms from the muscles or peripheral sensory receptors (mechanoreceptors, nociceptors, etc.) to the spine and brain (Collins, 2007; Carson and Buick, 2020). NMES has also been utilized as a neurorehabilitative tool to reduce muscle atrophy, and to improve muscle tone and motor function in patients with paralysis after stroke (Knutson et al., 2015; Yang et al., 2019) or spinal cord injury (SCI) (Patil et al., 2015). The working principle of rehabilitative NMES is based on the following: (1) the direct effect on muscle tone and (2) the activation of receptors and sensory axons that send afferent volleys to the sensorimotor cortex, after being processed by spinal networks and subcortical structures.

Numerous studies using functional magnetic resonance imaging (fMRI) and near-infrared spectroscopy (NIRS) have investigated the activity produced by NMES in different brain structures (Kampe et al., 2000; Blickenstorfer et al., 2009; Iftime-Nielsen et al., 2012; Schürholz et al., 2012; Wegrzyk et al., 2017). These works demonstrated that the brain activity is proportionally increased with the applied stimulation intensity (Backes et al., 2000; Smith et al., 2003; Schürholz et al., 2012). Recent experiments have also shown that peripheral stimulation can modulate corticospinal excitability, measured by motor evoked potentials (MEP) (Chipchase et al., 2011; Veldman et al., 2014). As the stimulation intensity increases, there is a progressive recruitment of more afferent receptors (i.e., cutaneous mechanoreceptors, muscle spindles, and Golgi tendon organs) that modulate spinal and cortical circuits to a different extent (Maffiuletti et al., 2008; Bergquist et al., 2011; Golaszewski et al., 2012). It has been proven that the afference provided by muscle spindles and Golgi tendon organs due to muscle contraction travels through the spinal cord to the somatosensory cortex and can directly project to the motor cortex (Carson and Buick, 2020). Therefore, the presence or absence of muscle contraction elicited by NMES has a direct impact on somatosensory cortex and, indirectly, on motor cortex excitability (Sasaki et al., 2017). The neural excitability depends on the modulation of nervous structures, such as spinal networks, involved in the afferent transmission from the stimulated muscle to the brain.

Although the effect of intensity on cortical activation and corticospinal excitability has been investigated, the dose or energy of the stimulation has not attracted much attention and might play a pivotal role in modulating the activity of sensory regions. There is evidence showing that the number of peripheral stimulation pulses (i.e., dose) over time and the inter-pulse interval influence ongoing neural oscillations and have a neuromodulatory effect on the somatosensory cortex affecting indirectly corticospinal connectivity (Nitsche and Paulus, 2000; Schaworonkow et al., 2018; Zrenner et al., 2018). The nervous system maintains its excitability within an equilibrium range through adjustments derived from the history of neuronal activity, preventing excessive inhibition or facilitation (Pozo and Goda, 2010). Prolonged periods of stimulation-induced excitability/inhibition have been shown to activate homeostatic plasticity mechanisms that drive the system toward a more inhibited/excited state, reducing the effects of the stimulation (Gamboa et al., 2010; Andrews et al., 2013). Further, a progressive perceptual adaptation (or reduction of sensory responsiveness) has been evidenced after prolonged intervals of peripheral vibrotactile and electrocutaneous stimulation (Leung et al., 2005; Buma et al., 2007; Graczyk et al., 2018), indicating a neural compensation after a perturbation of the oscillatory neural system. Therefore, it could be conceivable that prolonged periods of NMES result in changes of neural excitability, resulting in a rebalance of the cortical response along time.

Although most of the studies so far have used fMRI and NIRS to track slow brain correlates, or TMS-induced MEPs to asses corticospinal excitability, electroencephalography (EEG) is a powerful neuroimaging technique with high temporal resolution that has been used to study sensorimotor processes (including sensory evoked potentials and rhythms) (Birbaumer et al., 1990; Buzsaki, 2006; Shibasaki and Hallett, 2006; Leodori et al., 2019). The sensorimotor oscillations that mainly comprise the rolandic alpha [(7–13) Hz] and beta [(14–30) Hz] rhythms have been thoroughly used to study cortical involvement during sensorimotor tasks (Ramos-Murguialday and Birbaumer, 2015; López-Larraz et al., 2018), being quantified as the event-related (de)synchronization (ERD/ERS) (Pfurtscheller and Lopes da Silva, 1999). Furthermore, it has also been used as a feature for neuromodulation of sensorimotor neural network via proprioception and haptics (Ray et al., 2020; Sebastián-Romagosa et al., 2020). To date, only a few studies have reported how oscillatory activity measured with EEG is modulated by NMES (Vidaurre et al., 2016, 2019; Tu-Chan et al., 2017; Corbet et al., 2018). Understanding this process is of great importance given the relevance of EEG and NMES for neurorehabilitation, especially for EEG-based neural interfaces. It is important to note that, although the neural response can be directly influenced by every stimulation parameter (e.g., frequency, pulse width, and pulse waveform), we focused on investigating the influence of intensity and dose of stimulation, since these are the parameters that are usually personalized for each patient.

In this work, we acquired EEG activity from 12 healthy participants during NMES of the wrist extensor muscles at three different intensities (two below and one above the motor threshold) to investigate the neuromodulatory effect of peripheral stimulation on the ongoing cortical oscillatory activity, while the subjects rested in a comfortable sitting position. Firstly, we wanted to confirm that as the intensity increases, there is a proportional neural excitation, probably resulting from the recruitment of more afferent fibers, which provide a greater projection of afferent volleys to the sensory cortex. Secondly, we wanted to assess if the dose of stimulation has an effect on the magnitude of neural excitation over time, expecting that after the stimulation has been provided during a prolonged time, the cortical responsiveness is lower.

## Materials and Methods

### Participants

Twelve right-handed healthy participants (four females, age = 27.5 ± 3.0 years, height = 176.3 ± 8.6 cm, weight = 69.8 ± 9.8 kg) were recruited to participate in the study. All of them signed an informed consent form. The experimental procedure was approved by the Ethics Committee of the Faculty of Medicine of the University of Tübingen (Germany).

**FIGURE 1:**
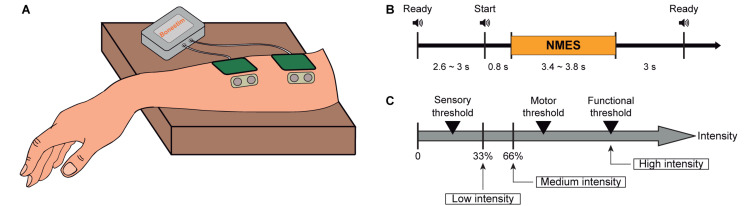
Experimental design and procedure. **(A)** Representation of the location of stimulation electrodes and sensors to measure muscle activity placed on right wrist extensors. **(B)** Timeline of the three phases included in each trial: preparation, NMES, and inter-trial period. **(C)** Determination of NMES intensities: low intensity as one third between the sensory threshold and motor threshold, medium intensity as two thirds between the sensory threshold and motor threshold, and high intensity as functional motor threshold.

Participants were asked to stay comfortably seated on a chair with their right arm resting on a side table and the hand hanging with the palm facing downwards. Neuromuscular electrical stimulation (NMES) electrodes were placed on the right-hand extensors (more detailed description in “*Neuromuscular Electrical Stimulation”* section), as described in Figure 1A. EEG and muscle activity of two sensors were recorded during the experiment. The electrical artifact recorded from the muscle sensors was used to align stimulation onset during the EEG signal processing.

### Experimental Design and Procedure

The main purpose of the experiment was to investigate NMES neuromodulatory effects (instantaneous and cumulative) on brain oscillatory activity. With this aim, we compared the afferent cortical activity generated by three different NMES intensities. Participants were passively stimulated, meaning that they were resting, and no volitional motor command was generated during stimulation. Each participant underwent one session consisting of nine blocks, each comprising 18 trials. One of the three NMES intensities was randomly assigned to each block (determination of the current intensities explained in “*Neuromuscular Electrical Stimulation”* section), resulting in three blocks per intensity. A ready cue was presented 2.6–3 s before the NMES interval, which had a random duration between 3.4 and 3.8 s. From the offset of the NMES to the next ready cue, a 3 s inter-trial period was introduced (see Figure 1B). Auditory cues announced the beginning of each interval. The time between blocks was used as breaks, lasting around 150 s (i.e., 2.5 min). The entire session including setup did not exceed 90 min.

### Data Acquisition

The electroencephalographic activity was recorded with a commercial 32-channel actiCAP system (Brain Products GmbH, Germany) and a monopolar BrainAmp amplifier (Brain Products GmbH, Germany). The recording electrodes were placed at FP1, FP2, F7, F3, Fz, F4, F8, FC3, FC1, FCz, FC2, FC4, C5, C3, C1, Cz, C2, C4, C6, CP5, CP3, CP1, CPz, CP2, CP4, CP6, P7, P3, P4, P8, O1, and O2, following the international 10/20 system. Ground and reference electrodes were placed at AFz and Pz, respectively.

Muscle activity of the right forearm of the participants was recorded by an MR-compatible BrainAmp amplifier (Brain Products GmbH, Germany) using two Ag/AgCl bipolar sensors (Myotronics-Noromed, Tukwila, Wa, United States). The sensors were placed laterally to the stimulation pads (see Figure 1A), using the right collarbone as ground. Both EEG and muscle activity were synchronously acquired at a sampling rate of 1,000 Hz.

### Neuromuscular Electrical Stimulation

A programmable neuromuscular stimulator Bonestim (Tecnalia, Serbia) was used to deliver the stimulation. The cathode (3 × 3.5 cm, self-adhesive electrode) was placed over the muscles involved in wrist extension (*extensor digitorum* and *extensor carpi ulnaris)*, at one third of the distance between the lateral epicondyle of the humerus and the Lister’s tubercle at the wrist. The anode (5 × 5 cm, self-adhesive electrode) was placed 5 cm distal to the cathode. To ensure the correct location of the electrodes, individually determined for each participant, stimulation above the motor threshold was applied until a complete wrist extension was induced.

The frequency of the NMES was set to 35 Hz, and the pulse width to 300 μs (Lynch and Popovic, 2008). The individual intensities for each subject were obtained by a scan of currents, starting at 1 mA and increasing in steps of 1 mA. The participants were asked to report the initiation of the following sensations: *(i)* tingling of the forearm (i.e., sensory threshold—STh), *(ii)* twitching of the fingers (i.e., motor threshold—MTh), and *(iii)* complete extension of the wrist (i.e., functional threshold—FTh). According to these thresholds, the three NMES intensities (two below and one above the motor threshold) were calculated, following the equations defined in the study of Smith et al. (2003). Low intensity was defined as one third between the STh and MTh; medium intensity as two thirds between the STh and MTh; and high intensity as the FTh (see Eq. 1–3 and Figure 1C). These intensities were kept constant throughout the experiment, and the complete extension of the wrist induced by functional stimulation was visually verified. None of the participants reported pain or any harmful effect due to the stimulation.

(1) Low⁢intensity=(MTh-STh)×0.33+STh

(2) Medium⁢intensity=(MTh-STh)×0.66+STh

(3) High⁢intensity=FTh

### Data Preprocessing and Analysis

#### Artifact Removal Procedures

One important limitation for the quantification of EEG activity during continuous stimulation is the contamination of the signals due to the electrical currents delivered to the body. The EEG is easily polluted by these currents, and artifact removal methodologies are essential to properly estimate cortical activation. With this aim, different techniques for contamination removal in invasive and non-invasive brain activity recordings have been proposed, such as interpolation, blanking, or linear regression reference (LRR) (Walter et al., 2012; Iturrate et al., 2018; Young et al., 2018). Blanking of the data is the most restrictive method as contaminated data are rejected and signals that could be of interest are neglected for further analysis. However, if the removal is implemented using hardware, the artifact has less influence on the recovery period of the amplifier, preventing it from being saturated and allowing the use of other methods to compensate for the missing data (Kent and Grill, 2012). Another approach is to linearly interpolate the corrupted data, connecting the last point before the artifact and the first point after the artifact. However, interpolation induces a bias in the estimation of power spectrum of the signals (Walter et al., 2012). LRR re-references the signals through weights that are assigned to each channel. The weights are calculated in a training block according to the noise of each channel generated by the electrical stimulation. This method effectively reduces artifacts, but it fixes the weights and assumes no changes in channel noise during the intervention. So far, the feasibility of this method has only been proven in invasive recordings (Young et al., 2018), in which impedances are less likely to change within sessions and are more similar among channels (Ball et al., 2009). Normally, impedances deteriorate and noise-influence increases throughout an EEG session, complicating the implementation of LRR in non-invasive recordings of brain activity. Therefore, we implemented an alternative two-step artifact removal method and demonstrated its feasibility. The raw EEG signals were pre-processed using custom-developed scripts in MATLAB (MathWorks, Natick, MA, United States).

##### Channel removal based on power-line noise

**FIGURE 2:**
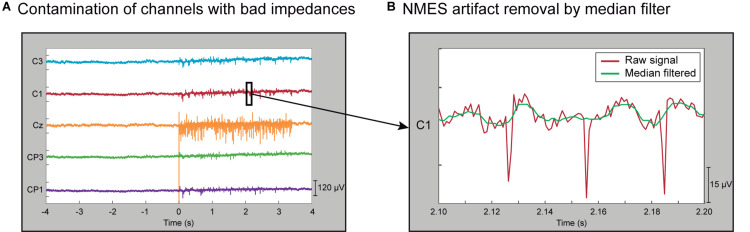
Characterization of contamination induced by NMES. **(A)** EEG of a representative trial showing non-contaminated channels (C3, C1, CP3, and CP1) and one contaminated channel (Cz) during high-intensity stimulation. Channels with bad impedances are more prone to be contaminated by electrical stimulation. **(B)** Zoom in 100-ms segment of a representative EEG trial in a non-contaminated channel that presents NMES artifacts (red line). The effect of stimulation artifacts is minimized by median filter (green line).

During an EEG session, particularly during setup and during periods between experimental blocks, special care is required to maintain EEG signal clean (i.e., raw data inspection and impedance check). However, our empirical experience shows that, sometimes, certain EEG electrodes present higher contamination due to the stimulation than others (see Figure 2A). These electrodes present broadband artifacts that impede further analyses even after applying the median filter preprocessing described below. We hypothesized that this effect might be due to degraded impedances, which occasionally deteriorate even when during the setup were set below 5 kOhm. Despite we did not store the impedances of each electrode to check this and discard the electrodes with high impedance, we ideated an automatized method to detect and discard them offline. This way, we could automatically eliminate contaminated channels without the human bias that would constitute a manual rejection.

**FIGURE 3:**
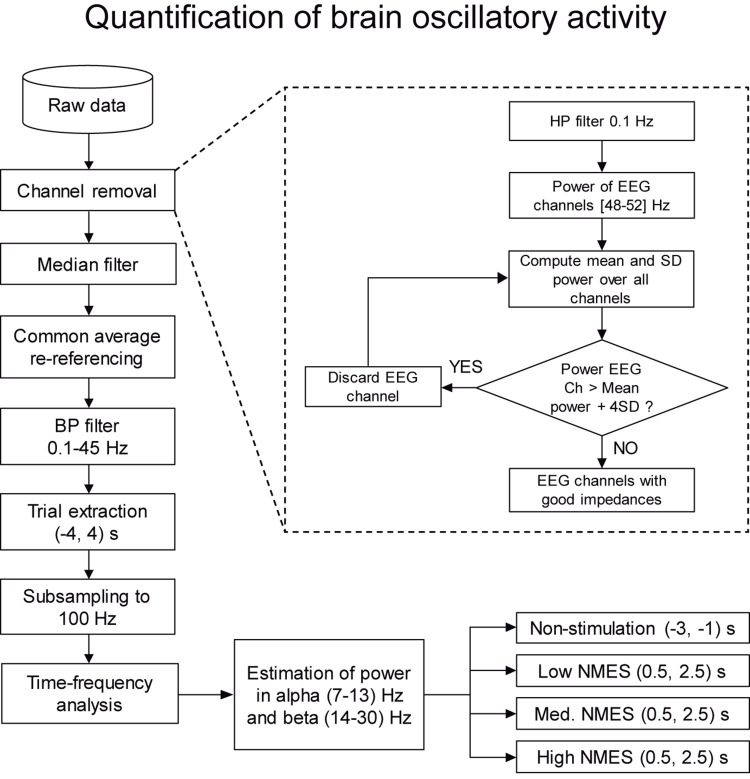
Flowchart with the steps for the quantification of brain oscillatory activity. The whole set of data is firstly preprocessed by a two-step procedure based on channel removal and median filtering. In this level, channels with bad impedances and artifacts due to electrical stimulation are removed. Then, the remaining clean data are filtered and divided into trials. Finally, power is estimated in alpha (7–13) Hz and beta (14–30) Hz bands for non-stimulation [(−3, −1) s] and NMES [(0.5, 2.5) s] intervals, being the baseline (−2.5, −1.5) s.

Having high impedance between the recording electrode and the skin resembles an open circuit, where the electrode behaves like an antenna and captures outside electric frequencies (like the power-line noise). Our method exploits this effect and identifies the EEG electrodes with unusually high power-line noise (50 Hz in Europe). Since all the electrodes should be approximately equally exposed to electromagnetic signals at 50 Hz, we assume that very high power at this frequency is an indirect indicator of high skin-electrode impedance. The procedure was applied block-wise, meaning that it was used to detect and remove contaminated channels within each individual EEG block. The EEG activity was high-pass filtered at 0.1 Hz with a 4th-order Butterworth. The power spectral distribution at 48–52 Hz was estimated using Welch’s method, averaging the periodogram of 1 s Hamming windows with 50% overlapping. The power mean and standard deviation (SD) of all the EEG channels were calculated from all trials in each block. Channels whose power was higher than 4 SD above the mean were discarded from that specific block. The remaining channels were used to re-compute the mean and SD. The procedure was iteratively repeated until no channels exceeded the rejection threshold (see Figure 3 dashed box, Supplementary Figures 1, 2, and Supplementary Table 1).

##### Median filtering for removal of electrical stimulation contamination

It is well known that applying electro-magnetic currents to stimulate the neural system can introduce undesired noise to the recordings. The NMES configuration used in this study introduces large peaks of short latency (∼5 ms) to the recorded EEG signal. Therefore, median filtering was used to minimize the NMES-induced artifacts (Insausti-Delgado et al., 2017). This filter is suited to eliminate high-amplitude peaks from a time series (Gallagher and Wise, 1981) and can remove the short-latency high-amplitude artifacts caused by the NMES. A sliding window of 10 ms was applied to the EEG signal in steps of one sample, providing as output the median value of each window. We selected a 10 ms window as it fully covers the electrical artifact. This filter produces a frequency-dependent attenuation that follows an exponential function from 0 to 100 Hz (i.e., the frequency with a period that completely fits within the 10 ms window), leading to low attenuation at low frequencies and a complete attenuation at 100 Hz (Supplementary Figure 3). With this window size, the attenuation of the signal at 10 Hz, 20 Hz, and 30 Hz is 1.28%, 4.89%, and 10.90% respectively. The relatively low attenuation at low frequencies makes this method suitable for analyzing alpha and beta sensorimotor oscillations. Figure 2B displays a zoomed segment of 100 ms of activity, showing the effect of the median filter on the stimulation artifacts.

### Quantification of Brain Oscillatory Activity

A common average reference (CAR) was applied to the EEG signals. The re-referenced signals were band-pass filtered at 0.1–45 Hz using a 1st-order Butterworth filter. Each block was trimmed down to (18×) 8-s trials, from -4 to + 4 s, being 0 the beginning of the stimulation. The trials were down sampled at 100 Hz, and those belonging to the same level of NMES intensity were pooled together.

The quantification of cortical activity was performed by evaluating the spectrum differences of the sensorimotor rhythms, by means of the alpha and beta event-related (de)synchronization (ERD/ERS), i.e., decrease or increase in power generated by an event compared to a baseline (Pfurtscheller and Lopes da Silva, 1999). Large ERD values (i.e., more negative power values) represent stronger cortical activation compared to baseline time interval, as it represents disinhibition/excitation of neural population activity (Ritter et al., 2009). For the quantification of brain activity, we used the FieldTrip toolbox^1^ for MATLAB. Time-frequency maps were calculated using Morlet wavelets in the frequency range from 1 to 45 Hz, with a resolution of 0.5 Hz. The power change was computed as the percentage of increase or decrease in power (i.e., ERS or ERD) with respect to the baseline [(−2.5, −1.5) s], where *P*_*j*_ represents the signal power at the *j*th sample, as described in Eq. 4.

(4) $$\text{ERD}/{\text{ERS}}_{j}\left(\%\right)=\frac{P_{j}-\text{Baseline}}{\text{Baseline}}\times 100$$

From the time-frequency maps, we calculated the sensorimotor averaged changes in power in alpha (7–13) Hz and beta (14–30) Hz bands for non-stimulation [(−3, −1) s] and NMES [(0.5, 2.5) s] intervals, using as baseline the (−2.5, −1.5) s interval (see Figure 3). The NMES period was defined as starting at 0.5 s to avoid potential bias and influence of the stimulation onset like event-related brain potentials (e.g., somatosensory evoked potential) at *t* = 0. For the topographical inspection and the descriptive analysis of the entire brain activity, all EEG channels were analyzed individually. For the quantitative analysis, we calculated the mean change in power of channels C1, C3, CP1, and CP3 (i.e., area over the sensorimotor cortex representing the right forearm, contralateral hemisphere to the stimulated limb), since we considered that averaged values over these electrodes could better quantify the overall changes in the sensorimotor areas.

### Statistical Analysis

The statistical tests were performed in IBM SPSS 25.0 Statistics software (SPSS Inc., Chicago, IL, United States) and MATLAB. We used the Shapiro–Wilk test to determine the normality of the data. Accordingly, a multivariate analysis of variance (MANOVA) for repeated measures was performed to find differences in the dependent variables, alpha and beta ERD/ERS, with NMES intensity (four levels: no stimulation, low-, medium-, and high-intensity stimulation) as within-subject factor. In order to determine the origin of the significant effect, *post hoc* tests with Bonferroni correction were performed.

In order to analyze whether NMES can induce a dose-effect, we studied the ERD/ERS changes over time. For that, we computed the alpha and beta ERD/ERS for each single trial (i.e., in Eq. 4, *P*_*j*_ was the alpha/beta power of each trial during the NMES period, and the baseline was calculated from the grand average of all the trials of each intensity). A linear regression was estimated for the ERD/ERS values over trials for the two frequency bands (i.e., alpha and beta) and the three NMES intensities (i.e., low, medium, and high). Correlation between ERD/ERS and sequence of trials were calculated using Pearson’s correlation coefficient to study stimulation effects over time.

## Results

### Effect of Artifact Removal

The pre-processing of the data eliminated satisfactorily the electrical noise contamination coming from the peripheral electrical stimulation. It reduced the effect of the artifacts to an extent that allowed us to perform EEG spectral analysis of the brain oscillatory activity.

**FIGURE 4:**
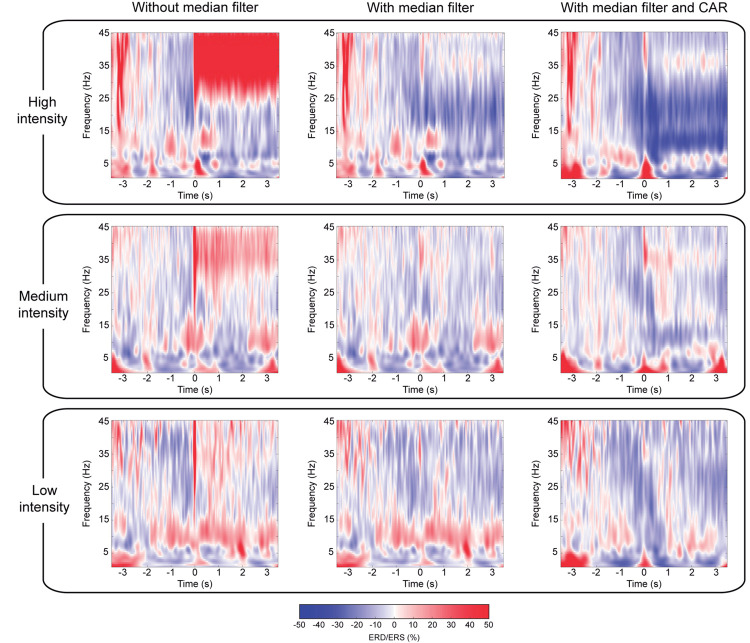
Comparison of cortical activation after median and spatial filtering. Time-frequency maps averaged over all participants, representing ERD/ERS, of the average of channels (C3, C1, CP3, and CP1) located over the contralateral sensorimotor cortex to the stimulated limb. Averaged time-frequency maps without median filter (left column), with median filter (center column), and with median and CAR filter (right column) after removal of contaminated channels. Rows show the different NMES intensities: high (upper row), medium (middle row), and low intensity (lower row). The percentage of ERD/ERS is computed according to the baseline (−2.5, −1.5) s. Time 0 s is aligned with the onset of the stimulation.

Channels with good impedances are also influenced by the electrical stimulation artifact that is introduced into the signal as large peaks. Figure 2B illustrates in a 100 ms segment of a representative trial how the median filter deals with these undesired artifacts. To prove the efficacy of the method, we focused on the worst-case scenario, as the contamination is larger for higher stimulation intensities. This effect can be observed in Figure 4, which depicts the EEG time-frequency activity at the different NMES intensities including artifacts and after the median and spatial filters are applied. The NMES generates an increase of power, or ERS, around 35 Hz (i.e., the stimulation frequency), which increases as the NMES intensity is incremented (Figure 4, left column). This power increase in high-beta/low-gamma band due to stimulation artifact was eliminated for all intensities after median filtering. Applying the median filter did not change the power in alpha and beta frequencies before the stimulation onset (*t* = 0 s), but eliminated the ERS during the stimulation period, minimizing the artifacts and revealing the alpha and beta modulation. The common average re-reference (CAR) after median filtering enhanced the power decrease of the bands of interest for every NMES intensity, as it does with non-contaminated EEG (Wolpaw et al., 2002). Regardless of the intensity delivered, the initiation of the stimulation generated time- and phase-locked activity (i.e., event-related potentials—ERP), presumably due to the sensory processing of the NMES (Reynolds et al., 2015). This can be seen as power increase at low frequencies (1–5 Hz).

### Influence of Stimulation Intensity on Cortical Activation

**FIGURE 5:**
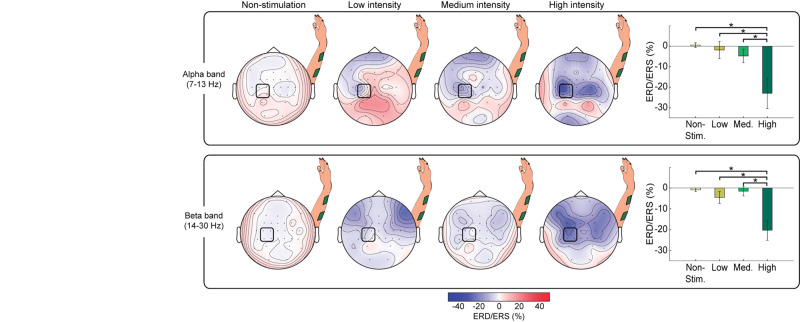
Comparison of cortical activation for different stimulation intensities for alpha and beta band EEG activity. Topographic maps, averaged over all participants, showing ERD/ERS of non-stimulation periods (−3, −1) s and NMES periods (0.5, 2.5) s belonging to each intensity (i.e., low, medium, and high) for alpha (upper row) and beta (lower row) frequency bands. Bar graphs show the mean percentage of ERD/ERS averaged from channels (C3, C1, CP3, and CP1) for each intensity and frequency band. The statistically significant differences between pairs are expressed with horizontal lines and stars. The percentage of ERD/ERS is calculated with respect to the baseline (−2.5, −1.5) s. The signals are processed using the two-step procedure (i.e., removal of contaminated channels and median filter) and a CAR.

To analyze the influence of stimulation intensity on cortical activation, we compared the changes in brain oscillatory activity in four conditions: non-stimulation, low-, medium-, and high-intensity stimulation (see topoplots of alpha and beta rhythms in Figure 5). Topographic maps of non-stimulation condition were calculated using the interval (−3, −1) s prior to the stimulation, while the other conditions were extracted from the interval (0.5, 2.5) s after stimulation onset. An increment of the stimulation intensity resulted in an increasing ERD (i.e., larger decrease in power) in both frequency bands over the sensorimotor cortex as expected (Backes et al., 2000; Smith et al., 2003; Schürholz et al., 2012), while occipital areas showed idling activity. At high-intensity stimulation, the sensorimotor cortex of both hemispheres presented a decrease of power, being more pronounced in the contralateral hemisphere, as demonstrated in previous work studying brain oscillatory signatures of motor tasks (Ramos-Murguialday and Birbaumer, 2015).

Our MANOVA analysis reflected a significant effect of intensity on alpha and beta ERD [*F*(6, 86) = 4.356, *p* = 0.001]. Rightmost panels in Figure 5 display the results of the *post hoc* comparisons. For both alpha and beta, there was a significantly higher ERD (i.e., more negative power values) induced by high-intensity NMES compared to rest (*p* = 0.004 for alpha, *p* < 0.001 for beta), to low-intensity NMES (*p* = 0.013 for alpha, *p* = 0.004 for beta), and to medium intensity (*p* = 0.045 for alpha, *p* < 0.001 for beta).

### Stimulation Dose-Effect

**FIGURE 6:**
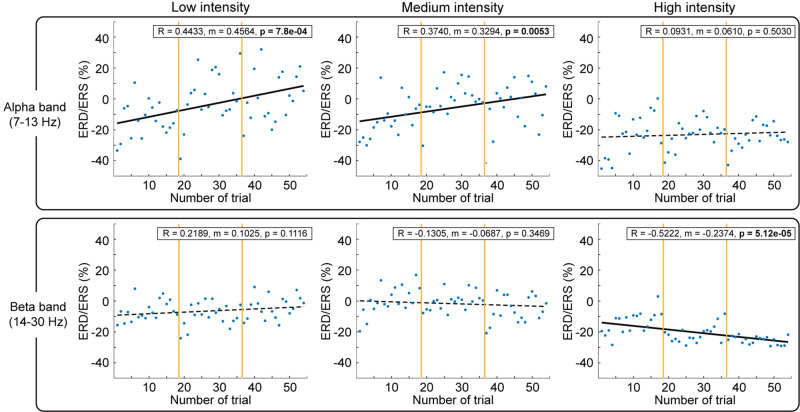
Comparison of cortical activation over trials for alpha and beta bands. The cortical activity during NMES period [(0.5, 2.5) s] quantified as ERD/ERS over the 54 trials for each stimulation intensity, divided into blocks by vertical yellow lines, and averaged for all the participants. The percentage of ERD/ERS is calculated according to the baseline (−2.5, −1.5) s. Different intensities are compared in columns: low (left), medium (middle), and high (right). Alpha (upper row) and beta (lower row) frequency bands are described. Significant correlations between ERD/ERS and sequence of trials over session are represented with black solid linear regressions.

To study the influence of stimulation dose on cortical activity, we computed the ERD/ERS of each single trial and performed a regression in time within session appending same stimulation intensity blocks in order of appearance (blocks of different intensities were presented randomly). Figure 6 shows the average ERD/ERS for all participants in both frequency bands during the 54 trials (3 blocks × 18 trials, see vertical yellow lines) for each intensity and a linear regression to fit them. We also performed regressions over time within each block with consecutive trials (see Supplementary Figure 4).

The first thing we observed is a clear modulation based on stimulation, reducing its variability with stimulation amplitude. Low and medium intensities caused a significant reduction of alpha ERD over time (*p* = 7.8e-4 for low; *p* = 0.0053 for medium). In contrast, high intensity caused a significant enhancement of beta ERD over time (*p* = 5.12e-5). Furthermore, as can be seen in Figure 6, we observed that in every block (separated by yellow vertical lines), there is a reduction of the ERD (increase of power plotted as linear regression in Supplementary Figure 4) progressively induced per trial from the first to the last trial. The first trial of the new block presented a larger ERD (decrease of power) in comparison to the ERD of the last trial of the previous block (irrespective of the stimulation intensity and order of the block within the session).

## Discussion

This study demonstrated the significant effects of artifacts, intensity, and dose of neuromuscular electrical stimulation (NMES) of the upper limb on the ongoing brain oscillatory activity recorded using EEG. First of all, we dealt with the issue of artifact removal to allow accurately estimating the cortical oscillatory activity. Recordings of brain activity are easily polluted, especially when electrical stimulation interacting with the nervous system is concurrently used. This contamination can negatively affect the signal-to-noise ratio, covering the brain activity. Our findings evidenced that the median filter enhanced the detection of sensorimotor oscillatory activity after removing stimulation artifacts.

After the EEG data was cleaned, especially of NMES-induced artifacts, we analyzed the modulation of alpha and beta oscillations produced by the stimulation. Power suppression, or desynchronization, of these frequencies has been associated with cortical excitation, whereas synchronization reflects a state of inhibition (Klimesch et al., 2007). During high-intensity NMES, the induced desynchronization in alpha and beta was significantly larger than during stimulation at low or medium intensities or no stimulation. While below-motor-threshold stimulation intensities only activate cutaneous mechanoreceptors (e.g., Pacinian corpuscles and Merkel disks) and sensory axons, stimulation above the motor threshold also recruits proprioceptive receptors (e.g., muscle spindles, Golgi tendon organs, and joint afferents) (Maffiuletti et al., 2008; Bergquist et al., 2011; Golaszewski et al., 2012). It has been proposed that muscle spindles transmit inputs to the spinal cord and can directly influence the motor cortex (M1) through the area 3a, while the projections from area 3b activated by cutaneous feedback to M1 are less likely to happen (Carson and Buick, 2020). We can therefore assume that high-intensity NMES leads to higher neural excitation, probably by recruiting a larger number of receptors derived from muscle contractions, in addition to the cutaneous and sensory fiber afference that is also engaged in sensory-threshold stimulation, and that the recruitment of muscle spindles results in activation of M1 via area 3a of the somatosensory cortex (S1) (Schabrun et al., 2012; Carson and Buick, 2020). These results of intensity-dependent brain activation are in line with corticomuscular responses (Sasaki et al., 2017), metabolic responses recorded by functional magnetic resonance imaging (fMRI) (Backes et al., 2000; Smith et al., 2003) and near-infrared spectroscopy (NIRS) (Schürholz et al., 2012), which demonstrated a direct quantitative association with stimulation intensity. Noteworthy, our results showed that the cortical activity measured with EEG, quantified as event-related (de)synchronization (ERD/ERS), during low and medium intensities was not significantly different to no stimulation. This suggests that below-motor-threshold NMES might not recruit enough afferent fibers to induce significant cortical modulation and that more afference (probably through muscle contraction, proprioception due to the movement, and a larger number of sensory fibers recruited) is required to transmit more information that reaches the brain and is measurable in the EEG at the analyzed frequencies.

It is well known that during voluntary movement, a stronger cortical activation is seen in alpha than in beta (López-Larraz et al., 2014; Ramos-Murguialday and Birbaumer, 2015). Such modulation in alpha and beta cortical activities has been related to the control of top-down and bottom-up neural processes, suggesting its role in the integration of motor task preparation and execution with movement-related sensory feedback. However, this balance between alpha and beta rhythms is altered in the absence of top-down regulation. Passive mobilizations (e.g., bottom-up transmission) exhibit stronger beta band activity compared with active movements, indicating the relationship of this frequency band with proprioception without volitional muscle contraction (Alegre et al., 2002; Ramos-Murguialday and Birbaumer, 2015), and thus the inhibitory effect of top-down neural control in this frequency. In this study, the peripheral electrical stimulation at the functional intensity level not only generated passive movement of the limb but also non-volitional contraction of the forearm muscles. This stimulation intensity induced a significant brain activation in beta frequency band, suggesting that this change might be comprised by two components: proprioception and afference of muscle contraction (without volition) through Golgi tendons and muscle spindles. Therefore, our results give a hint of the relevance of beta rhythm on bottom-up neuromodulation (i.e., afferent neural excitation). In agreement with previous studies, we can speculate that there might be differences in the afferent modulation between passive movements and functional electrical stimulation (Francis et al., 2009; Iftime-Nielsen et al., 2012). Whereas NMES over the motor threshold recruits afferent axons from muscle spindles, Golgi tendon organs, and cutaneous receptors (Maffiuletti et al., 2008; Bergquist et al., 2011; Golaszewski et al., 2012), Golgi tendon organs are less sensitive to passive movements and discharge less (Paillard and Brouchon, 1968; Purves et al., 2004), and the firing rate of the muscle spindles is muscle lengthening dependent (Chye et al., 2010). However, it cannot be concluded whether our functional NMES induces stronger beta activity than passive movements since we did not include the latter condition in our experimental protocol.

We tracked changes of the EEG sensorimotor oscillatory activity and evidenced that NMES induced a dose-effect on the activation patterns over time. To minimize the muscle fatigue due to NMES, we defined a low stimulation frequency as described in the literature (Gregory et al., 2007; Barss et al., 2018) and let non-stimulation periods to the muscle tissue to recover. However, we are aware that our results might be limited by muscle fatigue and that the stimulation parameters (e.g., pulse width) could be optimized to recruit central pathways and reduce fatigability (Collins, 2007). Regardless of the stimulation intensity applied, both alpha and beta bands presented a short-term reduction of ERD (reduction of brain excitatory effect of NMES) between consecutive trials within a block, showing that the ERD response of a specific trial depended on the previous stimulation. This reduction of ERD vanished at the beginning of every new block, demonstrating the ability of the sensorimotor oscillations to reset its excitability after the ∼2 min inter-block period. However, the overall activity throughout the session depicts that long-term effects survive temporary resets and exhibits a dose-effect over time, suggesting a conditioning effect. Despite the discontinuities due to inter-block pauses that could limit the significance of our results, we observed different long-term modulatory responses between sensorimotor oscillations in alpha and beta bands conditioned by the stimulation intensity.

For sensory-threshold intensities (i.e., low and medium), the power in alpha band was significantly reduced throughout the session, suggesting a habituation effect (Leung et al., 2005). NMES at sensory-threshold recruits cutaneous receptors and sensory axons (without eliciting any muscle contraction or movement) that activate spinal pathways and provide sensory afference to the brain (Maffiuletti et al., 2008). It has been evidenced that alpha band is relevant for information processing of attention and awareness (Händel et al., 2011; Klimesch, 2012), and we speculate that the repeated activation of functionally irrelevant sensory afference (i.e., cutaneous and sensory axon afference in the absence of movement) results in inhibition of the sensorimotor oscillations in alpha. A progressive habituation or desensitization (i.e., reduction of perceived sensation) of sensory perception is also presented after prolonged vibrotactile and electrocutaneous stimulation (Graczyk et al., 2018), which is slower at high stimulation intensities (Buma et al., 2007). This desensitization might be caused by a hyperpolarization of axon membranes (i.e., increasing membrane inhibition) controlled by the activity of Na-K pump that prevents the membrane from excessive excitation due to the repetitive electrical stimulation (Kiernan et al., 2004; Nodera and Kaji, 2006). One can hypothesize that the desensitization of sensory perception and habituation of alpha band might be connected somehow. However, we cannot conclude whether attention shifts over time could also induce the habituation in alpha oscillations, although non-significant fMRI responses have been reported in S1 when comparing different attention levels combined with sensory-threshold stimulation (Backes et al., 2000).

The modulation of beta power due to stimulation at functional-threshold intensity incremented with time, indicating an excitatory effect on the sensorimotor neural network. This result contrasts with what we initially expected, that is, a lower cortical responsiveness after prolonged stimulation. Beta oscillations have been related to the neural transmission from the primary motor cortex to the muscles and back to the motor cortex, via afferent sensory pathways, spinal cord, and somatosensory cortex (Aumann and Prut, 2015; Khademi et al., 2018). This closed-loop neural network provides the sensorimotor cortex with information of movements, comprising the muscles and joints. NMES at functional intensity recruits proprioceptive receptors (e.g., muscle spindles, Golgi tendon organs, and joint afferents) in addition to cutaneous mechanoreceptors (Maffiuletti et al., 2008; Golaszewski et al., 2012). One plausible explanation of our results is that the activation of this larger number of receptors keeps the aforementioned loop working and results in higher excitability of the network over time. Humans are constantly performing motor tasks, and movement drives our behavior and has driven our nervous system development. We can speculate that functionally relevant afferent information excites the sensorimotor cortex, while afferent information not related to movement (i.e., sensory axon activation and mechanoreception due to low and medium NMES) might not be considered “relevant” and therefore is omitted, suppressing neural excitability (Schabrun et al., 2012). Consequently, stimulation above the motor threshold has been proposed to result in improvements of motor function due to the secondary afference coming from muscle contractions and joint feedback, in addition to the primary sensory afference by NMES (de Kroon et al., 2005). All this highlights the different effects when muscle contraction and proprioception together with afference of sensory receptors enrich afferent activity (probably due to their ecologically relevant role in sensorimotor function), indicating that habituation or attention shift during a movement is less likely to occur due to its functional role in sensorimotor function.

To the best of our knowledge, this is the first time a sensorimotor cortical facilitation and inhibition effect due to NMES has been measured using EEG and characterized as presenting significant intensity- and dose-effects, which occurred in a short period of time. We particularly focused on these stimulation parameters because in NMES-based rehabilitative interventions, other parameters are usually fixed, while intensity and dose are modified. EEG is a widely used neuroimaging tool and, thanks to its good temporal resolution, has a great potential in the context of neurorehabilitation, especially in EEG-guided neural interfaces. Understanding how NMES parameters, such as intensity and dose, can modulate the excitability of cortical oscillations measured by EEG could be clinically relevant. For instance, NMES could be used for regulating rolandic alpha, since motor recovery has been related to an enhancement of this activity (Tangwiriyasakul et al., 2014; Ray et al., 2020), or could be integrated in rehabilitative neural interfaces that are controlled by sensorimotor oscillations.

We are aware that to be able to deepen into the neural mechanisms involved in the transmission of volleys from the muscle to the brain, we would need additional and more complex measurements to characterize the afferent pathways (including different reflexes, cortical and subcortical MEP, or sensory evoked potentials). Investigating how intensity and dose of NMES can affect other EEG features, such as phase, connectivity, or low-frequency oscillations, could also complement the here presented results. Further work should disclose whether more functional afferent activity (including proprioception and muscle contraction) is needed to increase functional plasticity and modulate sensorimotor function (e.g., corticomuscular synaptic efficacy, cortico-cortico functional connectivity, etc.). The effect of ongoing activity and other stimulation parameters (i.e., pulse width, pulse form, frequency, and energy) need to be carefully studied (Kampe et al., 2000; Wegrzyk et al., 2017), probably based on computational neuroscience and bioelectromagnetic modeling, to understand their effect in excitatory and inhibitory mechanisms. Nevertheless, the presented results shed some light onto the neuromodulatory effects that can be investigated and exploited using NMES.

### Study Limitations

First, the main limitation of the current study is that the utilized neuroimaging tool only allowed us to characterize excitatory changes of the sensorimotor cortex. However, we cannot conclude that other subcortical structures, such as the spinal cord, might also be modulated by the stimulation (Mang et al., 2011) and, in turn, affect the inputs arriving to the cortex. Nevertheless, we assume that our experimental paradigm ensured a constant state. Second, among the wide range of stimulation parameters (i.e., current intensity, frequency, pulse width, waveform, etc.) that could be tuned to modulate the neural excitability, we focused on the effect of current intensity, and the results of this study are limited by the stimulation parameters selected. Although there is evidence showing that using larger pulse widths and higher frequencies to recruit central pathways through sensory axons might have a greater neural impact (Collins, 2007; Lagerquist and Collins, 2010), we defined our stimulation parameters within the ranges that have already been used for rehabilitation of motor function in paralyzed patients (Quandt and Hummel, 2014; Knutson et al., 2015; Carson and Buick, 2020). Third, despite following the experimental protocols of Backes et al. (2000) and Smith et al. (2003), the absence of arm fixation and objective measurements of wrist extension makes it difficult to ensure that the different trials had the same effect on the neuromuscular system over time, especially as muscle fatigue progresses. However, we assumed that our experimental protocol regarding electrode size, careful electrode placement, and visual inspection for clean and consistent wrist extension over time minimized the variability of the results. Fourth, not including the conditions of passive wrist extension (without stimulation) and high-intensity NMES with the arm fixed in our experimental design also limits our results. Since we cannot isolate the afference contribution of the joint movement, muscle contraction, and sensory axon activation, it is not possible to disclose which components contributed more to the regulation of brain activity during NMES. Finally, due to the differences in the afferent projections to the cortex from distinct muscle groups (Mang et al., 2011), we cannot assume that the interpretations taken from our results could be generalized to other muscles. Altogether, further research should address these issues to better understand the neuromodulatory effects of NMES.

## Data Availability Statement

The data employed in the current study cannot be publicly shared. In case of interest in the data, it could be made available upon reasonable request contacting the senior researcher (ander.ramos-murguialday@uni-tuebingen.de) and the corresponding author (ainhoa.insausti-delgado@uni-tuebingen.de).

## Ethics Statement

The studies involving human participants were reviewed and approved by the Ethics Committee of the Faculty of Medicine of the University of Tübingen. The patients/participants provided their written informed consent to participate in this study.

## Author Contributions

AI-D, EL-L, JO, and AR-M made the study concept and design, were responsible for data interpretation, and revised the manuscript. AI-D, EL-L, and JO were responsible for data acquisition. AI-D and EL-L performed data analysis and wrote the draft. All authors contributed to the article and approved the submitted version.

## Conflict of Interest

EL-L is currently employed by a commercial company (Bitbrain). This company had no role in study design, data collection and analysis, decision to publish, or preparation of the manuscript. The authors declare that the research was conducted in the absence of any commercial or financial relationships that could be construed as a potential conflict of interest.

## Footnotes

1 http://fieldtriptoolbox.org/
