# Supplementary material for: Intensity and Dose of Neuromuscular Electrical Stimulation Influence Sensorimotor Cortical Excitability
Source: Front Neurosci. 2021 Jan 15;14:593360. doi: 10.3389/fnins.2020.593360 (PMC7845652; doi:10.3389/fnins.2020.593360)
Supplement: Supplementary file 1 [file Data_Sheet_1.pdf]

Supplementary Material

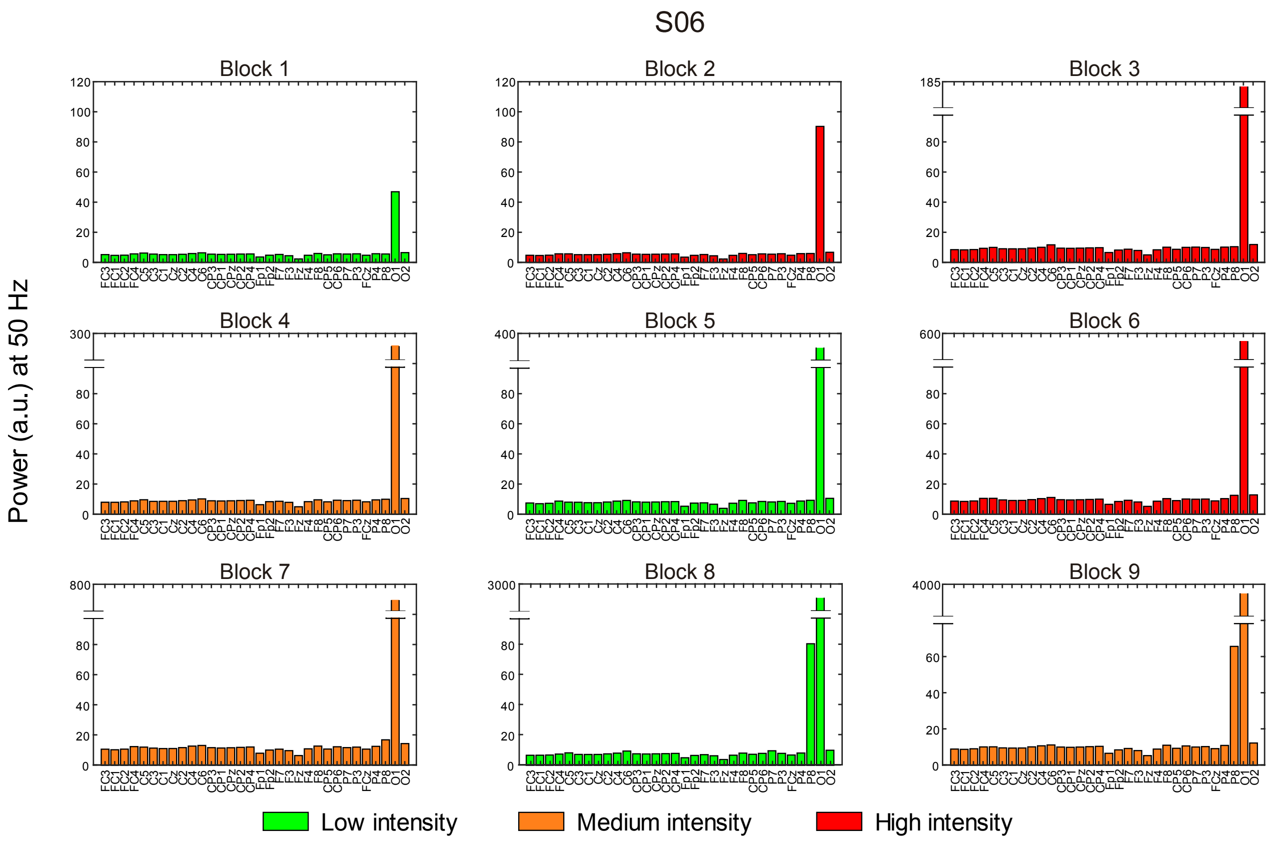

Supplementary Figure 1 **Estimation of power-line noise for channel removal.** The barplots represent the power-line noise in each EEG channel for every block of a representative subject. The green, orange and red color of the bars is associated with low, medium and high NMES intensity, respectively. High power-line noise was as an indicator of bad impedance and bad electrode-skin conductivity. We proposed a method based on the removal of the channels that exceeded the mean EEG power + 4 standard deviations. In this particular subject, O1 was rejected from all the blocks, and P8 from block 8 and block 9**.**

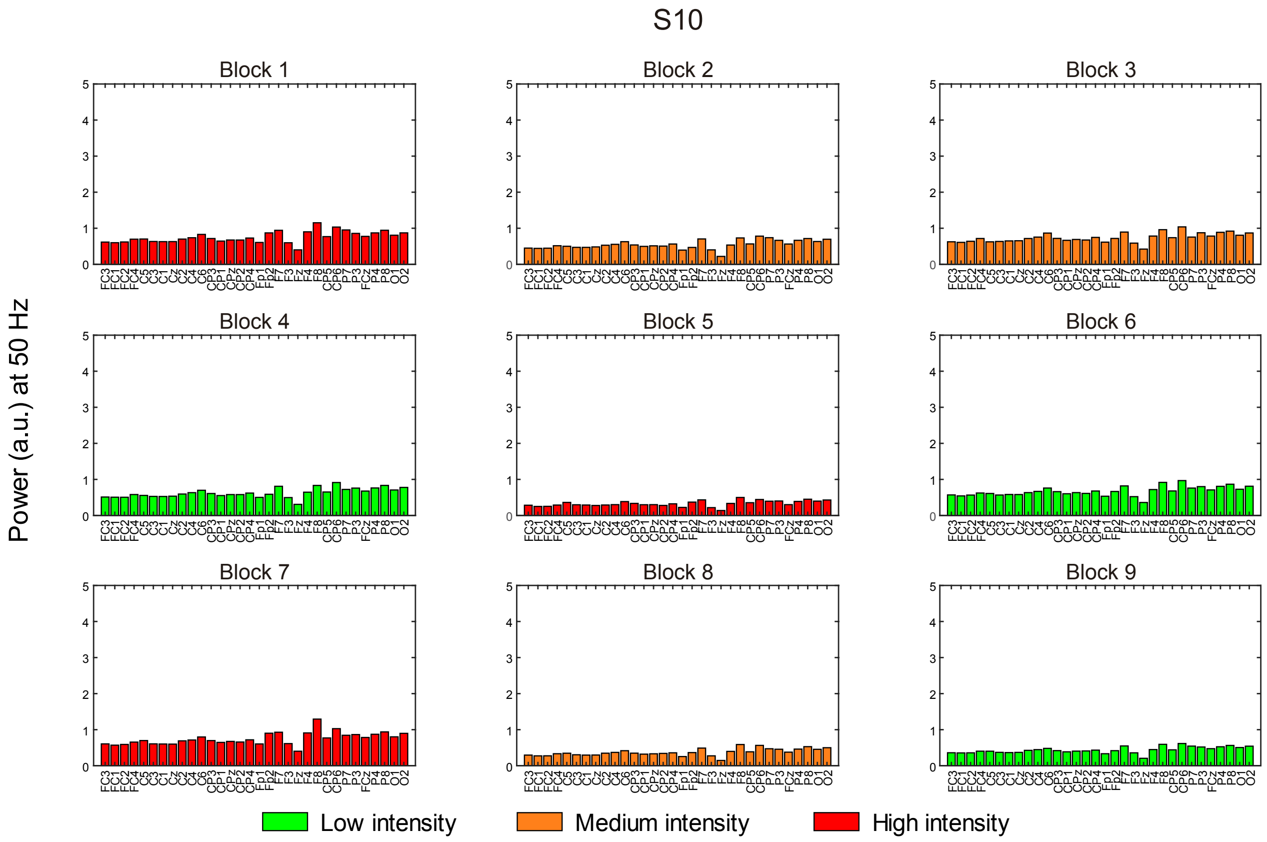

Supplementary Figure 2 **Estimation of power-line noise for channel removal**. The barplots represent the power-line noise in each EEG channel for every block of a representative subject. The green, orange and red color of the bars is associated with low, medium and high NMES intensity, respectively. High power-line noise was as an indicator of bad impedance and bad electrode-skin conductivity. We proposed a method based on the removal of the channels that exceeded the mean EEG power + 4 standard deviations. In this particular subject, all the channels in every block satisfied the inclusion criteria.

	Block 1	Block 2	Block 3	Block 4	Block 5	Block 6	Block 7	Block 8	Block 9
Subject 1	FP1, FC3	FP1	FP1	FP1	FP1	FP1	FP1	FP1, F7	FP1, C5
Subject 2	FC1	FC1	FC1, FC2	FC2	F4, FC2	FP2, F4, FC2	FP2, FC2	FP2, F4, FC2	FP1, FP2, F4, FC2
Subject 3									FP1, FP2
Subject 4			Cz	Cz	Cz	Cz	Cz	Cz	Cz
Subject 5		Fz	Fz	Fz	Fz	Fz		Fz	
Subject 6	O1	O1	O1	O1	O1	O1	O1	P8, O1	P8, O1
Subject 7									
Subject 8									
Subject 9									
Subject 10									
Subject 11					Fz	Fz	Fz	FP1, FP2, Fz	
Subject 12		Fz	Fz	Fz				Fz	

Supplementary Table 1 **Channel removal according to the power-line noise.** We proposed a method based on the removal of the channels that exceeded the mean EEG power + 4 standard deviations. The table shows the list of the channels that exceeded the rejecting threshold for every subject in each block**.**

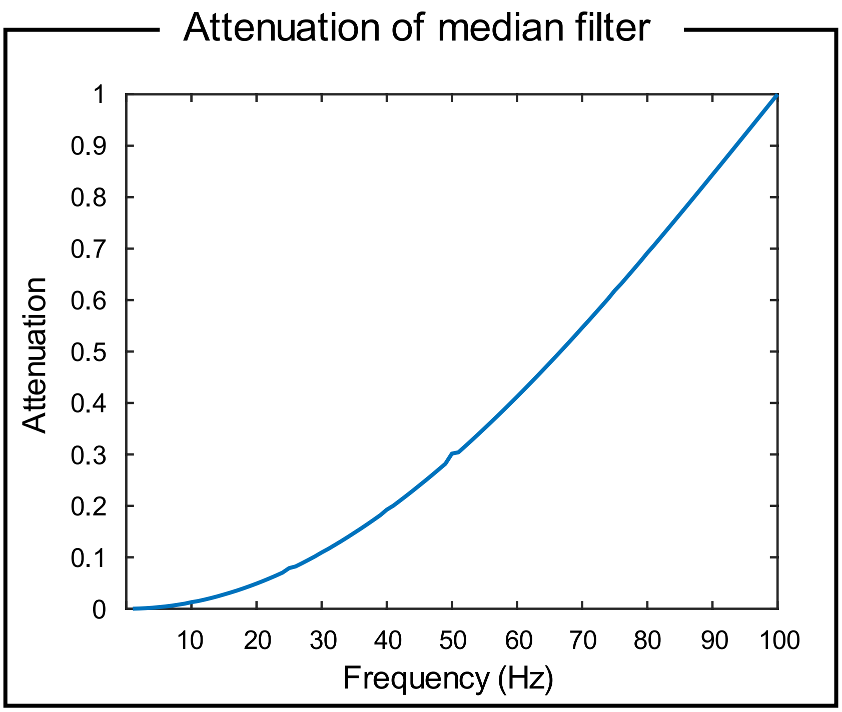

Supplementary Figure 3 **Attenuation of median filter.** The median filter induces an exponential attenuation of the signal, which is frequency dependent. This filter attenuates from 0 (no attenuation) to the inverse of the selected window length of the filter; in our case the window of 10 ms produces a total attenuation at 100 Hz. As an example, the attenuation at 10 Hz, 20 Hz and 30 Hz is 1.28%, 4.89% and 10.90%, respectively.

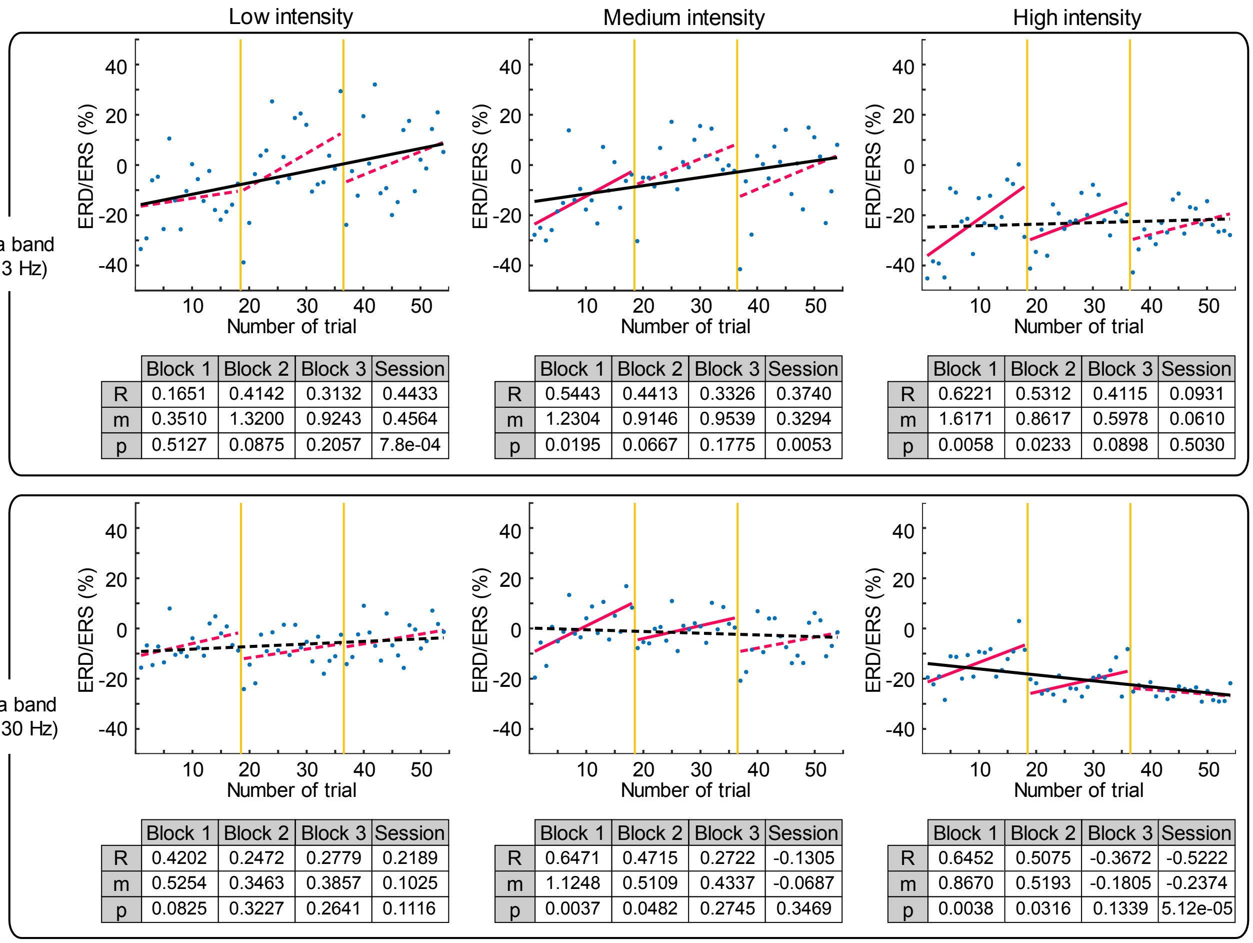

Alpha band (7-13 Hz), Low intensity

	Block 1	Block 2	Block 3	Session
R	0.1651	0.4142	0.3132	0.4433
m	0.3510	1.3200	0.9243	0.4564
p	0.5127	0.0875	0.2057	7.8e-04

Alpha band (7-13 Hz), Medium intensity

	Block 1	Block 2	Block 3	Session
R	0.5443	0.4413	0.3326	0.3740
m	1.2304	0.9146	0.9539	0.3294
p	0.0195	0.0667	0.1775	0.0053

Alpha band (7-13 Hz), High intensity

	Block 1	Block 2	Block 3	Session
R	0.6221	0.5312	0.4115	0.0931
m	1.6171	0.8617	0.5978	0.0610
p	0.0058	0.0233	0.0898	0.5030

Beta band (14-30 Hz), Low intensity

	Block 1	Block 2	Block 3	Session
R	0.4202	0.2472	0.2779	0.2189
m	0.5254	0.3463	0.3857	0.1025
p	0.0825	0.3227	0.2641	0.1116

Beta band (14-30 Hz), Medium intensity

	Block 1	Block 2	Block 3	Session
R	0.6471	0.4715	0.2722	-0.1305
m	1.1248	0.5109	0.4337	-0.0687
p	0.0037	0.0482	0.2745	0.3469

Beta band (14-30 Hz), High intensity

	Block 1	Block 2	Block 3	Session
R	0.6452	0.5075	-0.3672	-0.5222
m	0.8670	0.5193	-0.1805	-0.2374
p	0.0038	0.0316	0.1339	5.12e-05

Supplementary Figure 4 **Comparison of cortical activation over trials for alpha and beta band**. The cortical activity during NMES period [(0.5, 2.5) s] quantified as ERD/ERS over the 54 trials divided into blocks by vertical yellow lines of each stimulation intensity, averaged for all the participants. The percentage of ERD/ERS is calculated according to the baseline (-2.5, -1.5) s. Different intensities are compared in columns: low (left), medium (middle) and high (right). Alpha (upper row) and beta (lower row) frequency bands are described. Significant correlations between ERD/ERS and sequence of trials over session are represented with black solid linear regressions. Within block significant correlations are displayed by magenta solid linear regressions. Tables show the correlation (R), slope (m) and p-value (p) for every block and session.
